# Expression of c-erb-B2 oncoprotein as a neoantigen strategy to repurpose anti-neu antibody therapy in a model of melanoma

**DOI:** 10.21203/rs.3.rs-4004491/v1

**Published:** 2024-04-03

**Authors:** Emmanuel M. Gabriel, Brian Necela, Deborah Bahr, Sneha Vivekanandhan, Barath Shreeder, Sanjay Bagaria, Keith L. Knutson

**Affiliations:** Mayo Clinic Florida; Mayo Clinic Florida; Mayo Clinic Florida; Mayo Clinic Florida; Mayo Clinic Florida; Mayo Clinic Florida; Mayo Clinic Florida

## Abstract

In this study, we tested a novel approach of “repurposing” a biomarker typically associated with breast cancer for use in melanoma. HER2/neu is a well characterized biomarker in breast cancer for which effective anti-HER2/neu therapies are readily available. We constructed a lentivirus encoding c-erb-B2 (the animal homolog to HER2/neu). This was used to transfect B16 melanoma *in vitro* for use in an orthotopic preclinical mouse model, which resulted in expression of c-erb-B2 as a neoantigen target for anti-c-erb-B2 monoclonal antibody (7.16.4). The c-erb-B2-expressing melanoma was designated B16/neu. 7.16.4 produced statistically significant *in vivo* anti-tumor responses against B16/neu. This effect was mediated by NK-cell antibody-dependent cell-mediated cytotoxicity. To further model human melanoma (which expresses <5% HER2/neu), our c-erb-B2 encoding lentivirus was used to inoculate naïve (wild-type) B16 tumors *in vivo*, resulting in successful c-erb-B2 expression. When combined with 7.16.4, anti-tumor responses were again demonstrated where approximately 40% of mice treated with c-erb-B2 lentivirus and 7.16.4 achieved complete clinical response and long-term survival. For the first time, we demonstrated a novel strategy to repurpose c-erb-B2 as a neoantigen target for melanoma. Our findings are particularly significant in the contemporary setting where newer anti-HER2/neu antibody-drug candidates have shown increased efficacy.

## Introduction

Since the early 2010s, immune checkpoint blockade has vastly improved clinical outcomes for patients with locally advanced and metastatic melanoma.^1–3^ However, up to 40 to 50% of these patients still die from melanoma or develop resistance to currently available immune checkpoint inhibitors.^4,5^ While there are other targeted agents available for melanomas with BRAF mutations, durability of their response is limited.^6^ Adverse events from immunotherapy or targeted therapies also limit their use, and there are populations of patients in whom immune checkpoint blockade is contraindicated, such as those with organ transplants.^7^ Therefore, novel therapies and approaches are needed to continue to improve outcomes for melanoma patients and overcome emerging mechanisms of resistance.

For melanoma and other cancers, there is much investigation into uncovering neoantigen biomarkers and an array of strategies to therapeutically target such neoantigens, including adoptive effector cell therapy and antigen-based vaccines.^8,9^ While the goal of these innovative approaches is to develop mainstream clinically effective treatments, this may not be the case for many early therapeutics or may take decades to become realized through the industrial pipeline.

While targeted agents specific for melanoma are limited (namely BRAF and MEK inhibitors), there are other cancers that have different targeted drugs with long-term durability. For primary, locally advanced, and metastatic HER2/neu + breast cancer, as examples, patients may benefit from anti-HER2 monoclonal antibodies (i.e., trastuzumab and pertuzumab) and the newer antibody-drug conjugates (ADCs), including ado-trastuzumab (T-DM1 or emtansine) and trastuzumab deruxtecan (T-DXd).^10,11^ These more contemporary agents have been shown to be effective even when the HER2 expression is low because the chemotherapeutic component of the ADC is delivered to the tumor targets in order to generate their effect.^12^

In this preclinical study, the goal was to determine whether the animal homolog of HER2/neu (namely the c-erb-B2 oncoprotein) could be “repurposed” as a neoantigen target for melanoma. It has been shown that human melanoma overexpresses HER2/neu in less than 5% of cases, and thus anti-HER2 therapies are not currently approved or used for melanoma.^13^ However, the rationale of this study was that if c-erb-B2 could be expressed in melanoma tumors, then anti-c-erb-B2 monoclonal antibodies would generate effective anti-tumor responses through recognition of repurposed anti-c-erb-B2 as a neoantigen target. Our hypothesis was that the use of a c-erb-B2-encoding lentivirus vector would be effective in expressing c-erb-B2 as a neoantigen for anti-c-erb-B2 monoclonal antibody and lead to effective anti-tumor responses in an orthotopic mouse model of melanoma.

## Results

### Construction of c-erb-B2 lentivirus vector and confirmation of transfected wild-type B16 (B16/neu).

**Figures 1:**
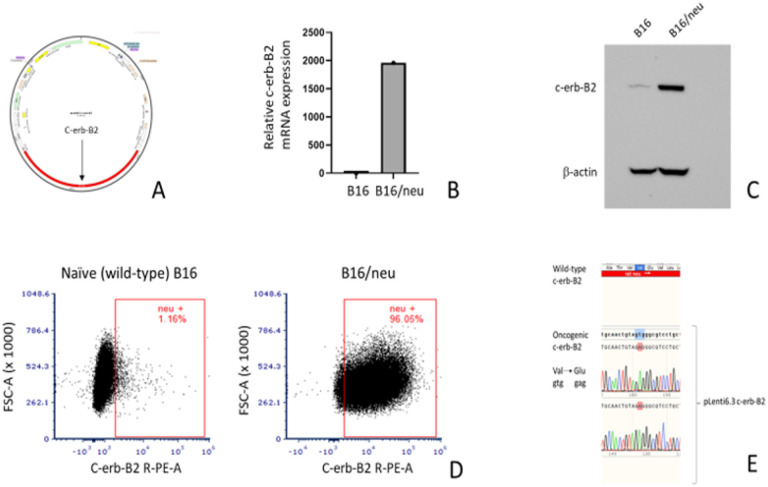
(A) pLenti6.3 c-erb-B2 construct showing the insertion of the rat c-erb-B2 (neu) gene into the lentivirus DNA. The c-erb-B2-expressing B16 melanoma cell line was designated B16/neu. c-erb-B2 expression by B16/neu was confirmed by qPCR (B), Western blot (C), and flow cytometry (D), which altogether showed high levels (>95%) of c-erb-B2 transcription and surface expression by the newly created B16/neu cell line. In contrast, naïve or wild-type B16 showed only about 1% c-erb-B2 expression. (E) The oncogenic variant of c-erb-B2 was inserted into pLenti6.3 and confirmed through gene sequencing, which demonstrated a valine to glutamine mutation. The oncogenic variant differs from wild-type c-erb-B2, which contains valine at this position.

Successful construction of the c-erb-B2 lentivirus (Fig. 1A) and transfection into naïve (wild-type) B16 melanoma cells were confirmed via q-PCR (Fig. 1B), Western blot (Fig. 1C), and flow cytometry (Fig. 1D). Original Western blots with a positive neu control using the c-erb-B2 expressing mouse cell line MMC (derived from mammary cells of female BALB/c mice) are included in the supplementary materials (Supplementary Fig. 1). Figure 1C shows a cropped version of this Western blot, which highlights the c-erb-B2 expression in our newly created c-erb-B2-expressing cell line compared to naïve B16. Similar to human melanoma, naïve B16 showed only approximately 1% endogenous c-erb-B2 (neu) expression on flow cytometry. This was significantly increased with pLenti6.3 c-erb-B2 transfection to over 95% surface expression of c-erb-B2. The c-erb-B2-expressing B16 was designated “B16/neu.” We also confirmed via gene sequencing that the c-erb-B2 insert represented the oncogenic variant of neu, as opposed to the wild-type phenotype (Fig. 1E).^14,15^ Expression of the oncogenic c-erb-B2 variant was important for our model in order to better replicate human breast cancer, where oncogenic HER2 overexpression is the target of anti-HER2 therapies.^16^

### Anti-neu monoclonal antibody (7.16.4) has no effect on B16/neu in vitro.

**Figure 2:**
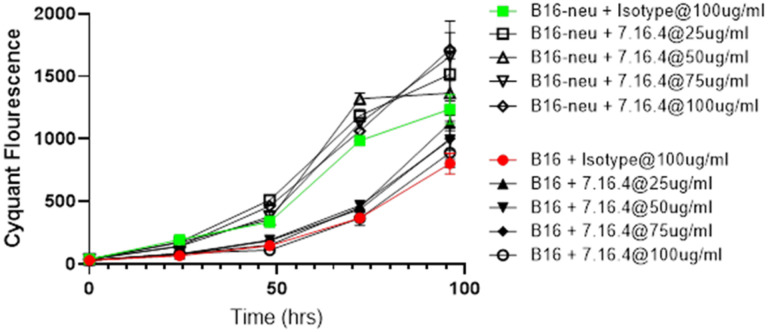
Naïve B16 and B16/neu were grown *in vitro*, and cell viability was assessed via the Cyquant assay. Overall, B16/neu showed higher cell viability at all time points compared to naïve B16. When the anti-c-erb-B2 monoclonal antibody 7.16.4 was added in increasing doses to B16/neu, no statistically significant effects were observed on cell growth at any of the tested doses.

Compared to naïve B16, the newly created B16/neu cell line showed increased growth and cell viability *in vitro* as measured by the Cyquant assay. This was observed throughout all time points up to 96 hours. When the mouse anti-rat c-erb-B2 monoclonal antibody 7.16.4 was added to the cell culture at increasing dose concentrations, there was no significant effect on B16/neu growth *in vitro* (Fig. 2). As expected, there was also no effect from 7.16.4 on naïve (non-c-erb-B2-expressing wild-type) B16.

Interestingly, the absence of effect of 7.16.4 on B16/neu was contrary to our group’s previous investigation of 7.16.4 in c-erb-B2 + mouse breast cancer (using the neu + MMC cell line).^17^ However, naïve B16 does not normally express oncogenic c-erb-B2 (as shown in Fig. 1D), and its oncogenicity is driven by other melanoma-specific mutations.^18,19^ Thus, neutralization of B16/neu growth by 7.16.4 *in vitro* was not expected.

### Anti-neu 7.16.4 generates in vivo responses against B16/neu.

To test our hypothesis that 7.16.4 could generate anti-tumor responses against B16/neu *in vivo*, B16/neu tumors were grown orthotopically within the dorsal skin of both male and female C57BL/6 mice. Although prior studies including our own have not demonstrated sex-based differences in B16 growth,^20,21^ we accounted for possible sex-based differences in the setting of the newly transfected c-erb-B2, which is most often associated with breast cancer cells in female subjects. Equal numbers of male and female mice were used for each experiment. Experiments were completed in triplicate to assess for reproducibility, and data were pooled for analysis.

For tumor subcuticular inoculation, 1 × 10^5^ B16/neu cells were injected in 0.1 ml of PBS with a 23-gauge needle. Treatments were initiated when tumors reached 5 mm^3^ in their longest dimension (approximately 7–10 days post-inoculation). As controls, naïve B16 at the same tumor inoculation dosage was included. The antibody 7.16.4 was administered intraperitoneally (ip) at 400 μg in 0.2 ml of PBS every 3 days until mice reached the predefined endpoints listed in the Methods. As an antibody control, isotype IgG2a was used at the same frequency and dosage ip (400 μg). Tumor measurements were taken every 5–6 days.

**Figure 3:**
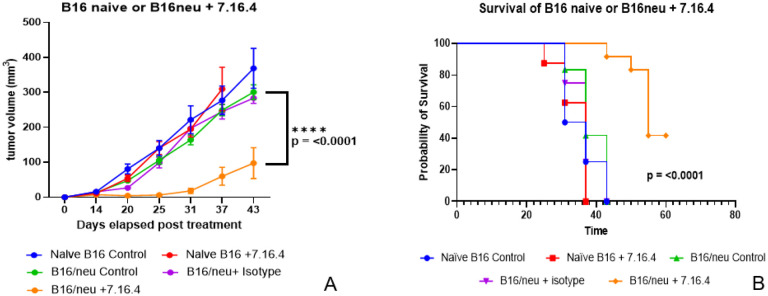
(A) 7.16.4 generated statistically significant responses on B16/neu growth compared to B16 and isotype (IgG2a) controls. (B) Survival was also significantly improved with 5/12 (41.2%) of C57BL/6 mice bearing B16/neu tumors achieving a complete clinical response and long-term survival.

Figure 3 shows the tumor growth (A) and survival analysis (B) for the control and experimental groups. As expected, there was no effect of 7.16.4 on naïve B16. There was slower tumor growth observed with the B16/neu controls (PBS or isotype) compared to naïve B16, but these differences were not statistically significant (p = 0.08 for PBS control, p = 0.09 for isotype control). When compared to isotype controls, B16/neu tumors treated with 7.16.4 showed statistically significant decreased tumor growth (p = < 0.000001) and improved survival with 5/12 (41.2%) tumors showing complete tumor response and regression (p = < 0.0001). Mice that obtained complete tumor response, of which 2 were female and 3 were male, did not have tumor volumes that exceeded 10 mm^3^ during the course of the experiment. This suggested that tumor growth kinetics beyond 10 mm^3^ was sufficient to overcome the anti-tumor effects of 7.16.4. Antibody therapy was stopped 60 days after tumor inoculation, and the mice with complete response showed long-term survival (over 90 days) and were then humanely euthanized.

### Anti-neu 7.16.4 responses are mediated by NK-cell antibody-dependent cell-mediated cytotoxicity.

Whereas 7.16.4 did not demonstrate a neutralizing effect on B16/neu *in vitro* (as shown in Fig. 2), we hypothesized that the mechanism by which *in vivo* c-erb-B2 expression facilitated anti-tumor responses from 7.16.4 (as shown in Fig. 3) was obtained by NK-cell antibody-dependent cell-mediated cytotoxicity (ADCC). To test our hypothesis, we included anti-NK cell monoclonal antibody (NK 1.1) at 200 μg injected every 3 days ip for C57BL/6 mice bearing B16/neu tumors. The NK 1.1 injections started 1 week before B16/neu inoculation to deplete NK cells prior to tumor growth and 7.16.4 treatment.

**Figure 4:**
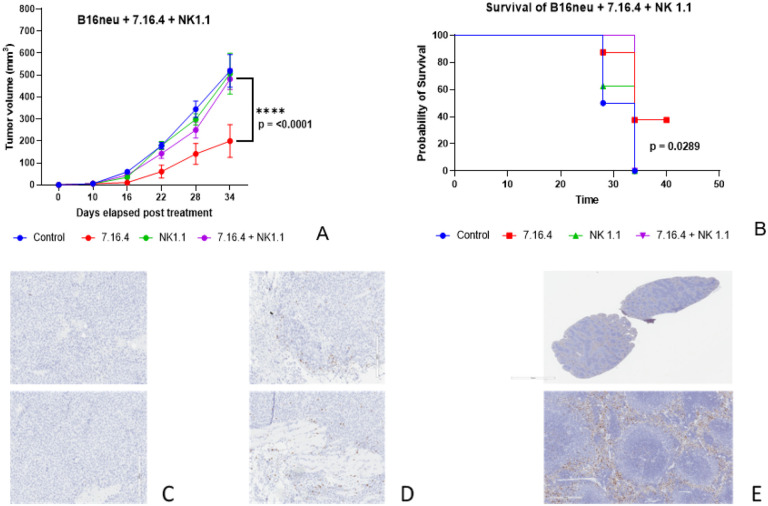
We hypothesized that 7.16.4 generated anti-tumor responses via NK-cell antibody-dependent cell-mediated cytotoxicity. The addition of anti-NK cell monoclonal antibody (NK 1.1) inhibited and essentially reversed the anti-tumor effects of 7.16.4 on both tumor growth (A) and survival (B). Resected tumors from mice treated with NK 1.1 showed little to no presence of NK cells on immunohistochemistry (C). In contrast, tumors resected from non-responders treated with only 7.16.4 showed a significantly higher number of stained NK cells on tumor sections (D), providing pathological evidence that NK 1.1 effectively eliminated NK cells from infiltrating into B16/neu tumors. IHC staining of NK cells within naïve spleens (obtained from non-tumor bearing C57BL/6 mice) was used as a control (E).

The addition of NK 1.1 inhibited the effect of 7.16.4 on B16/neu tumors (Fig. 4). Mice treated with NK 1.1 and 7.16.4 or NK 1.1 alone (control) showed similar growth rates (Fig. 4A) and survival (Fig. 4B) to the negative (PBS) controls. Equal numbers of male and female mice were used, and experiments were completed in triplicate. Again, mice treated with 7.16.4 only demonstrated superior statistically significant responses for both tumor growth (p = < 0.000001 compared to 7.16.4 + NK 1.1) and survival with 37.5% achieving complete clinical response and long-term survival (p = 0.0289).

Whole tumors were removed from mice treated with NK 1.1 +/− 7.16.4 and from non-responders that were treated with 7.16.4. Resected tumors were sectioned and analyzed by IHC for NK cells. Mice treated with 7.16.4 only (7.16.4 control) showed a qualitatively significant higher number of stained NK cells within the harvested B16/neu tumors (Fig. 4C) compared to mice treated with NK 1.1 +/− 7.16.4 (Fig. 4D), which showed essentially no NK cells within the tumor parenchyma. This provided pathological confirmatory evidence that NK 1.1 effectively inhibited NK cell activity via NK cell depletion, and that the *in vivo* anti-tumor mechanism of action of 7.16.4 was NK-cell ADCC. IHC for NK cells was also performed on normal (non-tumor bearing) C57BL/6 splenic tissue as a control, showing expectedly high levels of NK cells within the spleen parenchyma (Fig. 4E).

### In vivo transfection of naïve B16 melanoma tumors with c-erb-B2 lentivirus combined with 7.16.4 generates anti-tumor responses.

The presented experiments have thus far utilized B16/neu that was developed *in vitro* using our c-erb-B2 lentivirus (pLenti6.3 c-erb-B2). To better model human melanoma that does not normally express high levels of HER2/neu, we tested the *in vivo* injection of naïve B16 tumors with pLenti6.3 c-erb-B2. We hypothesized that *in vivo* transfection of naïve B16 tumors with pLenti6.3 c-erb-B2 would generate c-erb-B2 as a neoantigen target for 7.16.4 and allow for the repurposing of anti-c-erb-B2 (7.16.4) therapy.

To obtain the optimal dose of *in vivo* pLenti6.3 c-erb-B2, we tested a linear set of viral doses from 6 × 10^5^ pfu to 1 × 10^7^ pfu. These doses were injected into naïve B16 tumors when they grew to a minimum of 5 mm^3^. Virus was injected every 3 days. Aliquots of the different viral dosages were delivered in 50 μl PBS. At smaller tumor sizes (5–10 mm^3^), 1–2 injections into the tumor were performed. As tumors grew beyond 10 mm^3^, 3–5 injections were performed to cover as much of the tumor volume as possible. Virus injections were performed for a total of 4 intratumoral inoculations (or 12 days).

*In vivo* surface expression of c-erb-B2 as a neoantigen target for 7.16.4 was quantified by flow cytometry after resecting and processing the tumors. c-erb-B2 expression using this approach was shown to be dose dependent, with the highest dose (1 × 10^7^ pfu) yielding approximately 45% successful expression of c-erb-B2 *in vivo*. This was the selected dose of pLenti6.3 c-erb-B2 used in this set of experiments. As a virus control, normal or “blank” pLenti6.3 was used at the same intratumoral dose and schedule.

For C57BL/6 mice bearing naïve B16 (non c-erb-B2-expressing) tumors, 7.16.4 (400 μg ip) was added to pLenti6.3 c-erb-B2 after the first 2 doses (6 days) of virus (either pLenti6.3 control or pLenti6.3 c-erb-B2). Virus and 7.16.4 injections were given every 3 days until endpoints were met. Similar to our prior experiments, equal numbers of male and female mice were used, and experiments were completed in triplicate, again to meets standard for rigor and reproducibility.

**Figure 5:**
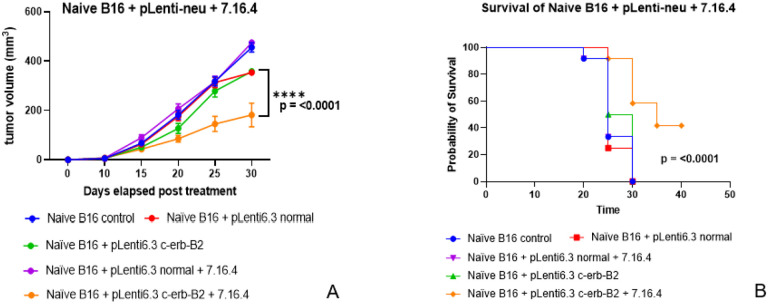
Naïve (wild-type) B16 tumors were inoculated with pLenti6.3 c-erb-B2 to generate c-erb-B2 as a neoantigen target for 7.16.4. The addition of 7.16.4 to pLenti6.3 c-erb-B2 resulted in statistically significant decreased tumor growth (A) and improved long-term survival (B), again with 41.2% of mice showing complete clinical response.

Figures 5A and B show the tumor growth and survival data, respectively. Similar to our experiments with B16/neu (derived *in vitro*) and 7.16.4, the *in vivo* inoculation of naïve (non-c-erb-B2) B16 tumors generated c-erb-B2 as a neoantigen target for 7.16.4. Naïve B16 tumors treated with pLenti6.3 c-erb-B2 and 7.16.4 showed statistically significant responses compared to isotype controls. These responses were demonstrated by decreased tumor growth (p = < 0.000001 ) and improved long-term survival (p = 0.0015). Similar to our experiments with B16/neu derived *in vitro*, our *in vivo* approach yielded a 41.2% complete clinical response (5/12 mice, 3 female and 2 male). These mice showed long-term survival to over 90 days. Again, tumors in these mice did not progress beyond 10 mm^3^ during the treatment period.

## Discussion

Herein, we demonstrated the feasibility and efficacy of repurposing c-erb-B2 as a neoantigen target via a c-erb-B2-encoding lentivirus vector. To our knowledge, this represents the first preclinical approach to utilize c-erb-B2 as a neoantigen in melanoma. Importantly, there were no sex-based differences even with c-erb-B2 most commonly being associated with breast cancer in females. c-erb-B2 expression was successfully generated both *in vitro* and *in vivo*, and anti-c-erb-B2 antibody produced highly significant responses with > 40% complete clinical response against B16, a highly aggressive melanoma tumor cell line. Responses appeared to be mediated via NK-cell ADCC, which is a well-established mechanism of antibody-mediated tumor killing.

Our preclinical model holds promise for future clinical relevance, particularly given the success of targeted anti-HER2/neu treatments. Anti-HER2/neu monoclonal antibodies (trastuzumab and pertuzumab) are part of the standard of care for all stages of breast cancer, including first line treatment for metastatic disease as well as for neoadjuvant treatment in resectable T2 primary tumors with or without lymph node metastases.^22,23^ Within the last decade, novel HER2-based antibody-drug conjugates (including ado-trastuzumab and trastuzumab deruxtecan) have been developed and shown to be even more effective than the monoclonal antibodies alone. These drugs have been approved for metastatic disease and adjuvant therapy, and in the future may be utilized in the neoadjuvant setting.^24–26^ Importantly, the anti-HER2/neu ADCs have been shown to effective in low HER2-expressing cancers because the chemotherapeutic component of the ADCs exerts its anti-tumor effect after targeted delivery to the HER2 + tumors, even if the HER2 expression is low.^27^ Altogether, this results in the bystander effect that is characteristic of ADCs, but not of monoclonal antibodies.^28^ Our model aims to take advantage of these currently available therapies and repurposes them for cancers that do not express HER2/neu as a therapeutic biomarker, such as melanoma. If successful, our novel approach may establish anti-HER2/neu therapies for cancers that have limited effective systemic options and open up known effective treatments to a larger number of cancer patients.

While we have demonstrated the effectiveness of our approach in a preclinical animal model, additional adjustments would need to be considered when translated to human therapy. We utilized the oncogenic (as opposed to wild-type) variant of c-erb-B2, comprising the entire gene sequence. Applying a similar approach to human therapy may increase the oncogenicity or metastatic potential of human cancers, which would be a counter-productive consequence of our approach. Thus, our future investigations will determine whether attenuated c-erb-B2 expression could generate similar responses. c-erb-B2 consists of superficial, transmembrane, and intracellular domains, which are altogether required to promote tumor growth.^29–31^ However, utilization of just the extracellular domain may be sufficient to repurpose anti-c-erb-B2 targeted therapy. The extracellular domain has been shown in breast cancer models to generate immune responses without increasing tumorigenicity, which occurs through signal transduction mediated by the transmembrane and intracellular components.^32^ Therefore, one of our future investigations will determine whether a virus vector encoded with only the extracellular c-erb-B2 sequence can yield similar anti-tumor responses. Refining our strategy in this manner may provide the most optimal combination of neoantigen recognition without increasing tumorigenicity, which will be critically important when translating our approach to human cancer studies.

Our chosen viral vector was the lentivirus pLenti6.3. While our recombinant plasmid pLenti6.3 c-erb-B2 was able to generate the B16/neu cell line *in vitro* (Fig. 1) and produce a high level of transfection and anti-tumor effect *in vivo* in naïve B16 tumors (Fig. 5), it is important to note that this vector does not result in cell lysis.^33,34^ As a non-oncolytic plasmid, pLenti6.3 has the advantage of limiting c-erb-B2 expression within the injected tumor targets and thereby minimizes neoantigen spread to bystander tissues or to distant tissues should the plasmid enter the bloodstream during intratumoral inoculations. While there is an intrinsically subjective component to the technique of intratumoral injections or vaccinations (where precision may vary from provider to provider), use of pLenti6.3 likely restricts c-erb-B2 expression to the site of injection.

We recognize that our data are early and are derived from a preclinical animal model of primary melanoma. Much of the morbidity and mortality for human melanoma stems from locally advanced, in-transit, and metastatic disease that are unresectable and unresponsive to current immunotherapies and targeted therapies.^35,36^ Whether our biomarker repurposing approach can be applied to regional or distant metastases was not addressed by these experiments. However, intratumoral injection of immunogenic viruses comprise a feasible, effective, and approved method for treating melanoma. Specifically, Talimogene Laherparepvec (T-VEC) is a herpes simplex virus oncolytic therapy that selectively replicates within melanoma and produces granulocyte macrophage colony-stimulating factor (GM-CSF) to enhance systemic anti-tumor immune responses.^37,38^ T-VEC can be injected into cutaneous melanoma tumors, but it can also be used for regional and distant metastases that can be accessed via image-guided percutaneous injections.^39^ Thus, there is an established precedent for intratumoral virus injections for melanoma at both cutaneous (superficial) and metastatic (deep) anatomical locations.

We also recognize that our strategy did not produce complete clinical responses in all animal subjects. However, the complete response rate was still quite high (> 40%) and appeared to be associated with tumor growth kinetics. If tumor growth was able to plateau (at approximately 10 mm^3^), then the success of our approach was significantly higher than if tumors grew more rapidly and outpaced any potential anti-tumor responses. This observation would represent another potential barrier of our approach to human clinical translation, where advanced disease tends to be larger or bulkier than small primary tumors. Nonetheless, our mouse model represents a promising starting point for our novel strategy and demonstrates proof of principle that c-erb-B2 can be repurposed as a neoantigen target for effective anti-c-erb-B2 therapies.

In conclusion, our approach to repurpose HER2/neu may be a timely, innovative strategy for melanoma and potentially other cancers that do not intrinsically overexpress oncogenic HER2/neu. Because of persistent limitations and emerging mechanisms of resistance to current therapies for melanoma, novel treatments are needed. In this study, the innovation of our approach is highlighted by the repurposing of contemporary effective therapies to malignancies for which they are not currently approved. Future preclinical studies are in development to determine whether our approach can be successful for regional or metastatic melanoma, larger primary tumors, and other cancers that do not normally express c-erb-B2.

## Materials and Methods

### Animals

Male and female C57BL/6 mice (6–8 weeks old) were purchased from the Jackson Laboratory (Bar Harbor, ME). Prior to use, mice were allowed to acclimate to the animal facility for 1 week.

Animal care and use were in accordance with institutional guidelines and approved under Mayo Clinic IACUC protocol A00005532–23: Expressing the oncoprotein neu on B16 melanoma as a therapeutic target for dynamic control of tumor vessels.

When animal experiments were completed, mice were humanely euthanized via a 30–70% per minute displacement of cage/chamber air with compressed CO2. This was confirmed with cervical dislocation per our IACUC approved protocol.

### Tumor cell line

B16 (subclone F10) melanoma cells were obtained from ATCC and authenticated. Cells were cultured in RPMI 1,640 (Roswell Park Memorial Institute Medium) supplemented with 10% FCS (fetal calf serum), 2 mM L-glutamine, 100 U/ml penicillin, 50 μg/ml streptomycin, and 50 μM β-mercaptoethanol (Invitrogen, Thermo Fisher Scientific, Carlsbad, CA).

### Reagents

FCS was obtained from Gemini Bioproducts (Woodland, CA). RPMI 1640, PBS, penicillin-streptomycin, L-glutamine, and β-mercaptoethanol were obtained from Life Technologies Inc. (Grand Island, NY). The monoclonal antibody 7.16.4, a mouse IgG2a antibody reactive with the rat neu oncogene-encoded p185 molecule, was obtained from Invitrogen (Thermo Fisher Scientific, Carlsbad, CA) and has been previously described by our group.^17^ The IgG2a isotype control antibody was also obtained from Invitrogen. Enhanced chemiluminescence reagents and enhanced chemiluminescence film were obtained from Amersham International (Oakville, Ontario, Canada).

### Construction of the B16 c-erb-B2 cell line (B16/neu)

A cDNA PCR amplicon corresponding to the full length c-erb-B2 gene was inserted into pLenti6.3 (Thermo Fisher Scientific, Carlsbad, CA) by NEBuilder^®^ HiFi seamless cloning (NEB, Ipswich, MA) in order to generate the plasmid pLenti6.3 c-erb-B2. High quantities of our lentivirus were generated by transfection of 293FT cells with pLenti6.3 c-erb-B2 and ViraPower^™^ packaging mix at a ratio of 1:3 using Lipofectamine 3000 per the manufacturer’s instructions (Thermo Fisher Scientific, Carlsbad, CA). Filtered fresh virus was added to naïve (wild-type) B16 cells at a 1:5 ratio in the presence of 6 μg/ml polybrene for 24 hr, and then media was replaced. At 72 hr post infection, infected B16 cells were selected with 10 μg/ml blasticidin for 2 weeks to generate the stable B16 c-erb-B2 cell line, which was designated B16/neu.

### Quantitative PCR

Two-step quantitative reverse transcriptase-mediated real-time PCR (qPCR) was used to measure the relative abundance of c-erb-B2 mRNA from equal amounts of cDNA (10 ng) of B16 and B16/neu using the TaqMan^™^ gene expression assay (Rn00566561_m1) and POLR2A (Mm00839502_m1) (Thermo Fisher Scientific, Carlsbad, CA). Data were normalized to the endogenous control POLR2A,^40^ and mRNA abundance was calculated using the ΔΔCT method.^40^

### Western blotting

Total cell lysates (50 μg) were resolved on Bolt 8% Bis–Tris Plus gels, which were electrophoretically transferred to a PDVF membrane with the iBlot2 apparatus (Thermo Fisher Scientific, Carlsbad, CA). Following 1 hour in blocking buffer [5% dry milk, TTBS (Tween Tris buffered saline, TBS + 0.05% Tween)], blots were stained with Cell Signaling (Danvers, MA) antibodies against HER2/c-erb-B2 (D8F12) and b-actin (13E5), followed by a goat anti-rabbit secondary antibody (7074). All blots were washed 3 times with TTBS after antibody staining. Bands were detected with the Amersham ECL Prime chemiluminescent system (Cytiva, Marlborough, MA) and visualized with film.

### Flow cytometry

For tumors, single cell suspensions were generated using a mouse tumor dissociation kit in combination with the gentleMACS^™^ Octo Desiccator (Miltenyi Biotec, Auburn, CA) per manufacturer protocol. Cell lines were harvested with a cell stripper dissociation reagent (Corning, Manassa, VA). Single cell solutions were washed twice in PBS and resuspended in 1X FACS buffer at 1 × 10^7^ cells/ml. Prior to staining, the monoclonal antibody 7.16.4 and IgG2a isotype control were first conjugated to DyLight^™^ 650 using the Lightning-Link antibody labeling kit (Novus Biologicals, Centennial, CO). Antibodies were added at 0.5 ug per 100 μl of cell sample and incubated for 30 min on ice, alongside a set of unstained samples. Samples were washed 4X with FACS buffer with centrifugations at 400G @ 5 min each. After the final wash, samples were resuspended in 0.5 ml FACS buffer with the viability dye SYTOX Green (1:1000), except for selected controls. Fluorescence was detected with a Attune NxT Cytometer (Thermo Fisher Scientific, Carlsbad, CA) and gated on viability. Plots and calculations were analyzed with the FCS Express software.

### Cell proliferation in vitro

Cells were plated at 3000 cells per well in a total volume of 200 μl in quintuple per condition/time point, using black, clear bottom 96 well plates. At each time point, cell proliferation was measured using the CyQUANT direct cell proliferation assay (C3501) according to manufacturer instructions (Thermo Fisher Scientific, Carlsbad, CA) and a SpectraMax M5 spectrophotometer (Ther Devices, San Jose, CA). For baseline (time = 0), cells were allowed to attach for 3 hr before measuring with the CyQuant assay. Background values of wells containing no cells (“no cell wells”) was subtracted from all data prior to plotting values.

### Assessment of tumor responses in vivo

Three perpendicular axes of the tumors were measured approximately every 5–6 days using external digital calipers (Control Co.). Tumor volume was calculated using the formula ½ × length × width × height. Mice that died or were euthanized due to morbidity, tumor ulceration, or tumor reaching the size endpoint (2,000 mm^3^) were classified as events. Measurements were performed by a lab member who was blinded to the treatment group. All *in vivo* experiments were performed in triplicate for replication. Group/treatment randomization was performed on the basis of cage position on the rack(s) within our vivarium. Cages were assigned a numerical designation. For each group, a cage was selected randomly from the pool of all cages. To establish blinding within each experiment involving tumor response in animals, tasks were completed by different members of the lab. These tasks included tumor cell preparation, tumor cell inoculation, treatment administration, and measurement of tumor response (tumor growth and survival). Following experiment completion, groups were unblinded in order to analyze the data.

### Immunohistochemistry

B16 tumor histologic sections were stained with standard hematoxylin and eosin and for NK cells [anti-mouse NK1.1 Monoclonal Antibody (PK136) —1:50 dilution, Invitrogen, ThermoFisher Scientific]. Stained sections were scanned with an Aperio Scanscope XT (Leica Microsystems Inc., Buffalo Grove, IL) and evaluated using the Aperio Spectrum software.

### Statistical Analysis

Cyquant cell viability and tumor volumes were assessed by a two-tailed Student t test. Tumor growth was assessed by ANOVA (GraphPad Prism, Version 6 software). Error bars represent standard errors of the mean unless otherwise noted. Standard Kaplan–Meier methods were used to perform the time to event analysis. For *in vivo* tumor growth experiments, a sample size of 12 mice per arm would yield at least 80% power to detect a difference of at least 1.3 standard deviations (std) in tumor volume, using a 2- sample t-test at the 0.05 significance level per experiment. For all experiments, values of p < 0.05 were considered significant.

## Data Availability

Data will be made available upon request. The corresponding author, Dr. Emmanuel Gabriel, can be contacted via telephone at 1-(904)-953–2523 and via email at Gabriel.Emmanuel@mayo.edu.

## Abbreviations

ADC: antibody-drug conjugates
ADCC: antibody-dependent cell-mediated cytotoxicity
ANOVA: analysis of variance
FCS: fetal calf serum
IHC: immunohistochemistry
KM: Kaplan-Meier
Ip: intraperitoneal
mm: millimeter
NK: natural killer
PBS: phosphate buffered saline
pfu: plaque-forming units
RPMI: Roswell Park Memorial Institute Medium
T-DM1: ado-trastuzumab
T-DXd: trastuzumab deruxtecan
TTBS: Tween Tris buffered saline
μg: microgram
μm: micrometer
μM: micromolar

## References

[1] Hodi F. S. Improved survival with ipilimumab in patients with metastatic melanoma. The New England journal of medicine 363, 711–723, doi:10.1056/NEJMoa1003466 (2010). 20525992 PMC3549297

[2] Ribas A. Pembrolizumab versus investigator-choice chemotherapy for ipilimumab-refractory melanoma (KEYNOTE-002): a randomised, controlled, phase 2 trial. The Lancet. Oncology 16, 908–918, doi:10.1016/s1470-2045(15)00083-2 (2015). 26115796 PMC9004487

[3] Robert C. Pembrolizumab versus Ipilimumab in Advanced Melanoma. The New England journal of medicine 372, 2521–2532, doi:10.1056/NEJMoa1503093 (2015). 25891173

[4] Wolchok J. D. Overall Survival with Combined Nivolumab and Ipilimumab in Advanced Melanoma. The New England journal of medicine 377, 1345–1356, doi:10.1056/NEJMoa1709684 (2017). 28889792 PMC5706778

[5] Marei H. E., Hasan A., Pozzoli G. & Cenciarelli C. Cancer immunotherapy with immune checkpoint inhibitors (ICIs): potential, mechanisms of resistance, and strategies for reinvigorating T cell responsiveness when resistance is acquired. Cancer cell international 23, 64, doi:10.1186/s12935-023-02902-0 (2023). 37038154 PMC10088229

[6] Grob J. J. Comparison of dabrafenib and trametinib combination therapy with vemurafenib monotherapy on health-related quality of life in patients with unresectable or metastatic cutaneous BRAF Val600-mutation-positive melanoma (COMBI-v): results of a phase 3, open-label, randomised trial. The Lancet. Oncology 16, 1389–1398, doi:10.1016/s1470-2045(15)00087-x (2015). 26433819

[7] Aguirre L. E., Guzman M. E., Lopes G. & Hurley J. Immune Checkpoint Inhibitors and the Risk of Allograft Rejection: A Comprehensive Analysis on an Emerging Issue. The oncologist 24, 394–401, doi:10.1634/theoncologist.2018-0195 (2019). 30413665 PMC6519766

[8] Hall M. S. Neoantigen-specific CD4(+) tumor-infiltrating lymphocytes are potent effectors identified within adoptive cell therapy products for metastatic melanoma patients. Journal for immunotherapy of cancer 11, doi:10.1136/jitc-2023-007288 (2023). PMC10565316 37802604

[9] Zeng T. Carrier-Free Nanovaccine: An Innovative Strategy for Ultrahigh Melanoma Neoantigen Loading. ACS nano 17, 18114–18127, doi:10.1021/acsnano.3c04887 (2023). 37695697

[10] von Minckwitz G. Trastuzumab Emtansine for Residual Invasive HER2-Positive Breast Cancer. The New England journal of medicine 380, 617–628, doi:10.1056/NEJMoa1814017 (2019). 30516102

[11] Hurvitz S. A. Trastuzumab deruxtecan versus trastuzumab emtansine in patients with HER2-positive metastatic breast cancer: updated results from DESTINY-Breast03, a randomised, open-label, phase 3 trial. Lancet 401, 105–117, doi:10.1016/s0140-6736(22)02420-5 (2023). 36495879

[12] Giugliano F., Curigliano G. & Tarantino P. HER2-low expression in breast oncology: treatment implications in the smart chemotherapy era. European journal of cancer prevention : the official journal of the European Cancer Prevention Organisation (ECP) 32, 149–154, doi:10.1097/cej.0000000000000781 (2023). 36693209

[13] Kluger H. M. Her2/neu is not a commonly expressed therapeutic target in melanoma -- a large cohort tissue microarray study. Melanoma research 14, 207–210, doi:10.1097/01.cmr.0000130874.33504.2f (2004). 15179190

[14] Guy C. T. Expression of the neu protooncogene in the mammary epithelium of transgenic mice induces metastatic disease. Proceedings of the National Academy of Sciences of the United States of America 89, 10578–10582, doi:10.1073/pnas.89.22.10578 (1992). 1359541 PMC50384

[15] Castagnoli L. Pathobiological implications of the d16HER2 splice variant for stemness and aggressiveness of HER2-positive breast cancer. Oncogene 36, 1721–1732, doi:10.1038/onc.2016.338 (2017). 27641338 PMC5447867

[16] Freudenberg J. A. The role of HER2 in early breast cancer metastasis and the origins of resistance to HER2-targeted therapies. Experimental and molecular pathology 87, 1–11, doi:10.1016/j.yexmp.2009.05.001 (2009). 19450579 PMC2735009

[17] Knutson K. L., Almand B., Dang Y. & Disis M. L. Neu antigen-negative variants can be generated after neu-specific antibody therapy in neu transgenic mice. Cancer research 64, 1146–1151, doi:10.1158/0008-5472.can-03-0173 (2004). 14871850

[18] Danciu C. A characterization of four B16 murine melanoma cell sublines molecular fingerprint and proliferation behavior. Cancer cell international 13, 75, doi:10.1186/1475-2867-13-75 (2013). 23890195 PMC3750233

[19] Nakamura K. Characterization of mouse melanoma cell lines by their mortal malignancy using an experimental metastatic model. Life sciences 70, 791–798, doi:10.1016/s0024-3205(01)01454-0 (2002). 11833741

[20] Simon S. R. & Ershler W. B. Hormonal influences on growth of B16 murine melanoma. Journal of the National Cancer Institute 74, 1085–1088 (1985). 3858578

[21] Gabriel E. M. Dynamic control of tumor vasculature improves antitumor responses in a regional model of melanoma. Sci Rep 10, 13245, doi:10.1038/s41598-020-70233-5 (2020). 32764623 PMC7413248

[22] Baselga J. Pertuzumab plus trastuzumab plus docetaxel for metastatic breast cancer. The New England journal of medicine 366, 109–119, doi:10.1056/NEJMoa1113216 (2012). 22149875 PMC5705202

[23] Schneeweiss A. Pertuzumab plus trastuzumab in combination with standard neoadjuvant anthracycline-containing and anthracycline-free chemotherapy regimens in patients with HER2-positive early breast cancer: a randomized phase II cardiac safety study (TRYPHAENA). Annals of oncology : official journal of the European Society for Medical Oncology / ESMO 24, 2278–2284, doi:10.1093/annonc/mdt182 (2013). 23704196

[24] Verma S. Trastuzumab emtansine for HER2-positive advanced breast cancer. The New England journal of medicine 367, 1783–1791, doi:10.1056/NEJMoa1209124 (2012). 23020162 PMC5125250

[25] Tolaney S. M. Adjuvant Trastuzumab Emtansine Versus Paclitaxel in Combination With Trastuzumab for Stage I HER2-Positive Breast Cancer (ATEMPT): A Randomized Clinical Trial. Journal of clinical oncology : official journal of the American Society of Clinical Oncology 39, 2375–2385, doi:10.1200/jco.20.03398 (2021). 34077270

[26] Mosele F. Trastuzumab deruxtecan in metastatic breast cancer with variable HER2 expression: the phase 2 DAISY trial. Nature medicine 29, 2110–2120, doi:10.1038/s41591-023-02478-2 (2023). PMC10427426 37488289

[27] Narayan P. US Food and Drug Administration Approval Summary: Fam-Trastuzumab Deruxtecan-nxki for Human Epidermal Growth Factor Receptor 2-Low Unresectable or Metastatic Breast Cancer. Journal of clinical oncology : official journal of the American Society of Clinical Oncology 41, 2108–2116, doi:10.1200/jco.22.02447 (2023). 36780610

[28] Nicolò E., Zagami P. & Curigliano G. Antibody-drug conjugates in breast cancer: the chemotherapy of the future? Current opinion in oncology 32, 494–502, doi:10.1097/cco.0000000000000656 (2020). 32657795

[29] Kanthala S., Mill C. P., Riese D. J. 2nd, Jaiswal M. & Jois S. Expression and purification of HER2 extracellular domain proteins in Schneider2 insect cells. Protein expression and purification 125, 26–33, doi:10.1016/j.pep.2015.09.001 (2016). 26363121 PMC4785095

[30] Lee J., Dull T. J., Lax I., Schlessinger J. & Ullrich A. HER2 cytoplasmic domain generates normal mitogenic and transforming signals in a chimeric receptor. The EMBO journal 8, 167–173, doi:10.1002/j.1460-2075.1989.tb03361.x (1989). 2565808 PMC400786

[31] Hudziak R. M. & Ullrich A. Cell transformation potential of a HER2 transmembrane domain deletion mutant retained in the endoplasmic reticulum. The Journal of biological chemistry 266, 24109–24115 (1991). 1684181

[32] Fendly B. M. The extracellular domain of HER2/neu is a potential immunogen for active specific immunotherapy of breast cancer. Journal of biological response modifiers 9, 449–455 (1990). 1979347

[33] Luis A. The Old and the New: Prospects for Non-Integrating Lentiviral Vector Technology. Viruses 12, doi:10.3390/v12101103 (2020). PMC7600637 33003492

[34] Hamilton A. M., Foster P. J. & Ronald J. A. Evaluating Nonintegrating Lentiviruses as Safe Vectors for Noninvasive Reporter-Based Molecular Imaging of Multipotent Mesenchymal Stem Cells. Human gene therapy 29, 1213–1225, doi:10.1089/hum.2018.111 (2018). 30101620

[35] Huang J. Global Incidence, Mortality, Risk Factors and Trends of Melanoma: A Systematic Analysis of Registries. American journal of clinical dermatology, doi:10.1007/s40257-023-00795-3 (2023). 37296344

[36] Gabriel E. & Skitzki J. The Role of Regional Therapies for in-Transit Melanoma in the Era of Improved Systemic Options. Cancers (Basel) 7, 1154–1177, doi:10.3390/cancers7030830 (2015). 26140669 PMC4586763

[37] Andtbacka RH, C. F., Amatruda T OPTiM: A randomized phase III trial of talimogene laherparepvec (T-VEC) versus subcutaneous (SC) granulocyte-macrophage colony-stimulating factor (GM-CSF) for the treatment (tx) of unresected stage IIIB/C and IV melanoma. 2013 ASCO Annual Meeting J Clin Oncol 31, 2013 (suppl; abstr LBA9008) (2013).

[38] Andtbacka R. H. Talimogene Laherparepvec Improves Durable Response Rate in Patients With Advanced Melanoma. Journal of clinical oncology : official journal of the American Society of Clinical Oncology 33, 2780–2788, doi:10.1200/jco.2014.58.3377 (2015). 26014293

[39] Ferrucci P. F., Pala L., Conforti F. & Cocorocchio E. Talimogene Laherparepvec (T-VEC): An Intralesional Cancer Immunotherapy for Advanced Melanoma. Cancers (Basel) 13, doi:10.3390/cancers13061383 (2021). PMC8003308 33803762

[40] Radonić A. Guideline to reference gene selection for quantitative real-time PCR. Biochemical and biophysical research communications 313, 856–862, doi:10.1016/j.bbrc.2003.11.177 (2004). 14706621

